# Capability of multiparametric MRI to detect diabetic nephropathy in high-risk patients with type 2 diabetes: a prospective preliminary study

**DOI:** 10.1186/s12880-026-02588-6

**Published:** 2026-07-20

**Authors:** Wei Mao, Xiaoqiang Ding, Caixia Fu, Bernd Kuehn, Dominik Nickel, Robert Grimm, Thomas Benkert, Mengsu Zeng, Jianjun Zhou

**Affiliations:** 1 Department of Radiology, Zhongshan Hospital, Fudan University, Shanghai Institute of Medical Imaging, 180 Fenglin Road, Shanghai, 200032 People’s Republic of China; 2 Department of Nephrology, Zhongshan Hospital, Fudan University, 180 Fenglin Road, Shanghai, 200032 People’s Republic of China; 3 MR Applications Development, Siemens Shenzhen Magnetic Resonance Ltd, Shenzhen, China; 4 Research & Clinical Translation, Magnetic Resonance, Siemens Healthineers AG, Erlangen, Germany

**Keywords:** Diabetic nephropathy, Intravoxel incoherent motion diffusion-weighted imaging, Diffusion kurtosis imaging, Arterial spin labeling, T1 mapping

## Abstract

**Background:**

Early diagnosis and treatment of diabetic nephropathy (DN) is important for improving prognosis of patients, but the noninvasive and reliable diagnostic tool is lacking. We aim to investigate the ability of multiparametric MRI for detecting early DN in high-risk patients with type 2 diabetes.

**Methods:**

Between June 2023 and October 2024, we prospectively recruited 80 patients(diabetic patients group, *n* = 42, 53.4 ± 10.0 years old; DN patients group, *n* = 38, 47.9 ± 10.9 years old) and 16 healthy volunteers(control group, 49.5 ± 9.5 years old). All participants were examined using multiparametric MRI(IVIM, DKI, ASL and T1 mapping). The true diffusion coefficient(D), perfusion fraction(f), mean diffusivity(MD), mean kurtosis(MK), renal blood flow(RBF), T1 values in renal cortex were measured. Renal cortical MRI parameters among 3 groups were compared by one-way analysis of variance. Correlation between estimated glomerular filtration rate(eGFR), 24-hour urine albumin(24 h-UA) and renal cortical MRI parameters was evaluated using Spearman correlation analysis. The diagnostic performances of MRI parameters compared with biochemical indexes for detecting early DN were assessed using receiver operating characteristic curves.

**Results:**

Renal cortical MRI parameters demonstrated statistically significant differences among 3 groups(*P* < 0.050). The eGFR, 24 h-UA significantly correlated with renal cortical MRI parameters(*P* < 0. 001). The areas under the curve(AUCs) for discriminating diabetic patients group from DN patients group were 0.706, 0.864, 0.732, 0.718, 0.910 and 0.718 for D, f, MD, MK, RBF and T1 values. AUCs of renal cortical f, RBF values were significantly larger than that of eGFR, serum creatinine, 24 h-UA, fasting blood glucose(*P* < 0.050).

**Conclusions:**

Intravoxel incoherent motion diffusion-weighted imaging and arterial spin labeling exhibited considerable promise as noninvasive tools for detecting DN in high-risk patients with type 2 diabetes.

## Background

The prevalence of diabetic nephropathy (DN) has been increasing globally. Globally, DN is regarded as one of the most prevalent causes of death and illness in patients with type 2 diabetes [1, 2]. When DN is diagnosed clinically, the injury to kidney caused by it is already intractable. Hence, early intervention and diagnosis are essential before DN progresses to an irreversible degree. Currently, DN is commonly diagnosed by detecting urinary microalbumin. Nonetheless, in the early stage of DN, the kidney’s own compensatory function often leads to a negative test result for urinary microalbumin [3]. While renal biopsy serves as the gold standard for detecting early DN, it is invasive and can raise the risk of complications [4]. Hence, a noninvasive and reliable method for detection of early DN is urgently needed.

Intravoxel incoherent motion diffusion-weighted imaging (IVIM-DWI), in which a biexponential model is applied to MR signal intensity decay, can assess water molecule diffusion and capillary perfusion of tissue [5]. Diffusion kurtosis imaging (DKI) is a functional MRI (fMRI) technique that evaluates non-Gaussian diffusion motion of water molecule in tissue [6]. Arterial spin labeling (ASL) designates inflowing blood as an endogenous contrast medium, which is used to evaluate microvascular perfusion in tissues [7]. T1 mapping, a quantitative MRI technique that has been shown to assess tissue fibrosis, might act as an alternative to renal biopsy [8]. Previous research has shown that IVIM-DWI, DKI, ASL and T1 mapping are capable of evaluating renal function and pathological injury in chronic kidney disease [9–12]. However, to our knowledge, a combination of these functional imaging tools to detect early DN is lacking. Hence, this study aimed to determine whether multiparametric fMRI parameters could act as noninvasive tools to identify early DN that requires further evaluation via renal biopsy in type 2 diabetes patients, including true diffusion coefficient(D) and perfusion fraction(f) (derived from IVIM-DWI), mean diffusivity(MD) and mean kurtosis(MK) (derived from DKI), renal blood flow(RBF) derived from ASL, T1 derived from T1 mapping.

## Methods

### Participants

The study protocol was approved by the institutional review board (protocol number: B2017-147R), and informed consent was obtained from all participants.

The consecutive type 2 diabetic patients (DP) who were suspected of having DN were enrolled in this prospective study between June 2023 and October 2024. Inclusion criteria were (a) a history of type 2 diabetes for more than 8 years, (b) no contraindication to MRI examination, (c) no other diseases affecting renal function (hypertension, hematologic system diseases, gout, etc.), (d) urine albumin level < 30 mg/24 h, (e) normal estimated glomerular filtration rate (eGFR ≥ 90 mL/min/1.73 m^2^). Exclusion criteria were (a) image quality was poor and affected the measurement, (b) presence of renal neoplastic lesions (diameter > 10 mm). We also recruited sixteen healthy volunteers as controls, and none of these volunteers had a history of diabetes, vascular diseases, or hypertension. Participants’ characteristics are summarized in Table 1.

**Table 1:** Participant characteristics

	Control group (*n* = 16)	Diabetic patients group (*n* = 42)		DN patients group (*n* = 38)	*p* value
Age (yr)	49.5 ± 9.5	53.4 ± 10.0	47.9 ± 10.9		0.058
Sex (male/female)	6/10	23/19	22/16		0.379
Serum creatinine (µmol/L)	47.7 ± 6.2	45.9 ± 10.8	49.9 ± 9.0		0.174
24 h-UA (mg/24 h)	17.7 ± 5.0	19.1 ± 7.1	20.4 ± 6.8		0.375
eGFR (mL/min/1.73 m^2^)	109.3 ± 8.5	111.0 ± 13.6	108.2 ± 9.6		0.549
FBG (mmol/L)	4.8 ± 0.9	6.5 ± 1.0	6.9 ± 1.2		< 0.001

eGFR: estimated glomerular filtration rate; 24 h-UA: 24-hour urine albumin; FBG: fasting blood glucose; DN: diabetic nephropathy

The 24-hour urine albumin (24 h-UA), fasting blood glucose (FBG) and serum creatinine (SCr) were assessed in every participant one day preceding their MRI examinations. We utilized the Chronic Kidney Disease Epidemiology Collaboration formula [13] for the calculation of eGFR.

### Magnetic resonance imaging

All participants underwent scanning with a 3 Tesla MRI system (MAGNETOM Prisma, Siemens Healthineers), which used an 18-channel body array coil and an integrated 32-channel spine coil as the receiver. All participants were required to fast for at least 6 h before MRI examinations. All relevant scan parameters are summarized in Table 2. Firstly, axial T1 and coronal T2 weighted images covering bilateral kidneys were obtained to characterize anatomy. Then, the research application sequences including IVIM, DKI, ASL and T1 mapping were performed sequentially. Zoomed field-of-view echo-planar imaging sequence was used to acquire multiple *b* DWI images for IVIM (*b* = 0, 25, 50, 80, 100, 150, 300, 500, 800, and 1000 s/mm^2^) and DKI (0, 500, 1000, 1500, and 2000 s/mm^2^) in coronal orientation. To minimize the impact of diffusion anisotropy, diffusion was encoded in 4 directions, which were then combined to form a trace-weighted images. To enhance the quality of DWI images, slight rotation of the field-of-excitation was performed during signal excitation, 2D in-plane motioncorrection was inline applied between b-values and averages with the inverse consistent deformable registration algorithm, and complex averaging to reduce Rician noise bias [14] were implemented. A free-breathing, pseudo-continuous arterial spin labeling (pCASL) sequence with the three-dimensional TGSE acquisition scheme was used to measure renal perfusion. Based on the formula used in a previous study [15], quantitative RBF maps originating from pCASL were generated inline. The labeling duration and post-labeling delay (PLD) were both set to 1500 milliseconds [9, 16, 17], with inflow arterial-blood suppression and background suppression being performed. Four parameters were configured as follows: the T1 of blood at 3 Tesla was set to 1.6 s, the arrival time of labeled blood was assumed to be 750 milliseconds, the inversion efficiency was set to 0.98, and the blood-tissue water partition coefficient was set to 0.9 mL/100 g. Retrospective motion compensation based on 3D rigid and elastic registration algorithms was applied before image averaging to mitigate respiratory motion artifacts in perfusion imaging. An inversion recovery SNAPSHOT-FLASH sequence with Look-Locker scheme was used to obtain T1 mapping. The number of inversion contrast images was configured to 32, and the waiting time between subsequently acquired slices was configured to 1000 milliseconds.

**Table 2:** MRI protocol parameters

	T1-WI	T2-WI	IVIM/DKI	ASL	T1 mapping
Sequence type	VIBE	HASTE	Zoomed-DWI	TGSE pCASL	Look-Locker SNAPSHOT-FLASH
Orientation	Transversal	Coronal	Coronal	Coronal	Coronal
Respiratory control	Breath-hold	Breath-hold	Free-breathing	Free-breathing	Breath-hold
TR/TE (msec)	3.3/1.3	1200/95	4000/54	5000/19.25	3/1.32
Voxel size (mm^3^)	1.2 × 1.2 × 3.0	1.2 × 1.2 × 4.0	2.0 × 1.7 × 5.0	4.7 × 4.7 × 5.0	2.0 × 2.0 × 8.0
FOV (mm^2^)	380 × 308	380 × 356	400 × 168	300 × 150	380 × 308
Matrix	320 × 240	320 × 272	200 × 100	64 × 32	192 × 153
Slices	60	30	15	16	3
Turbo factor	N/A	255	N/A	16	N/A
EPI factor	N/A	N/A	84	32	N/A
Fat suppression	SPAIR	SPAIR	FatSat	FatSat	Off
Acceleration factor	4 (CAIPIRINHA)	3 (GRAPPA)	2 (GRAPPA)	1	2 (GRAPPA)
Scan time	15 s	40 s	5mins54secs	5 min	17 s

VIBE: Volumetric Interpolated Breath-hold Examination; HASTE: Half-Fourier-Acquired Single-shot Turbo spin Echo; FOV: Field of View; N.A: Not Applicable; SPAIR: Spectral Attenuated Inversion Recovery; CAIPIRINHA: Controlled Aliasing in Parallel Imaging Results in Higher Acceleration; GRAPPA: GeneRalized Autocalibrating Partially Acquisitions

### Image analysis

IVIM-DWI and DKI parameter maps (D, f, MD, and MK) were generated using research application software Body Diffusion Toolbox (Siemens Healthineers). By fitting all 10 b values to the bi-exponential model, the D and f maps were generated [18]. By fitting all 5 b-values to a non-Gaussian diffusion model, we calculated the MD and MK [19]. Inline generation was performed for the RBF and T1 maps. An experienced radiologist drew the regions of interest (ROIs), while the first author analyzed all images; both were blinded to the participants’ clinical and pathologic information. Areas with artifacts, cysts, vascular structures, collecting system were excluded when drawing ROIs. Two ROIs (75–125 voxels each) that cover the full kidney cortex of each kidney were manually outlined on MRI parameter maps, specifically on two coronal planes near the renal hilum. For each kidney, renal cortical (RC) MRI parameters were obtained by averaging individual readings of cortical ROIs; afterward, the MRI parameters of the bilateral kidneys were averaged to be used in subsequent analyses. Moreover, an experienced radiologist randomly selected and analyzed MRI images of 30 participants, and intraclass correlation coefficients (ICC) (two-way random model, absolute agreement)were subsequently employed to evaluate the test-retest reliability of various fMRI parameters.

### Renal pathologic analyses

All DP underwent ultrasound-guided renal biopsy one day after they completed their MRI examination to determine whether DP have DN. Consistent with the method employed by Li et al. [20], we chose lower pole of right kidney as biopsy site. According to pathologic diagnostic criteria of DN [21], all pathologic specimens were analyzed by an experienced nephropathologist who was blinded to MRI information. Based on pathologic diagnosis results, the DP were divided into DP group and DN patients group.

### Statistical analyses

Statistical analyses were performed using SPSS (version 25.0; IBM), MedCalc (version 15.8; MedCalc Software), and Prism (version 5.0; GraphPad Software). Means ± standard deviation were used to represent quantitative variables, and categorical variables were depicted as the count of findings. To compare fMRI data between the right and left kidneys of all participants, the paired-sample t-test was employed. The difference in fMRI data between two groups (all patients and healthy volunteers) was evaluated using independent-sample *t*-test. The clinical indexes, laboratory indexes, and RC MRI parameters among the control, DP and DN patients groups were compared using nonparametric Kruskal-Wallis test or one-way analysis of variance, as appropriate. Tukey’s test was used to conduct post hoc multiple pairwise comparisons. Furthermore, Spearman correlation analysis was employed to assess the correlation between RC MRI parameters and the biochemical indicators (eGFR, 24 h-UA) of all participants. The Receiver operating characteristic (ROC) curve analysis was used to gauge the diagnostic ability of MRI parameters, in comparison with biochemical indexes, for distinguishing between DP and DN patients groups. We calculated the area under the ROC curve (AUC), specificity, sensitivity, and optimal cut-off values, then compared the AUCs by applying DeLong’s test [22]. Two-tailed *P* < 0.050 indicated a significant difference for all tests.

## Results

### Participant characteristics

**Fig. 1:**
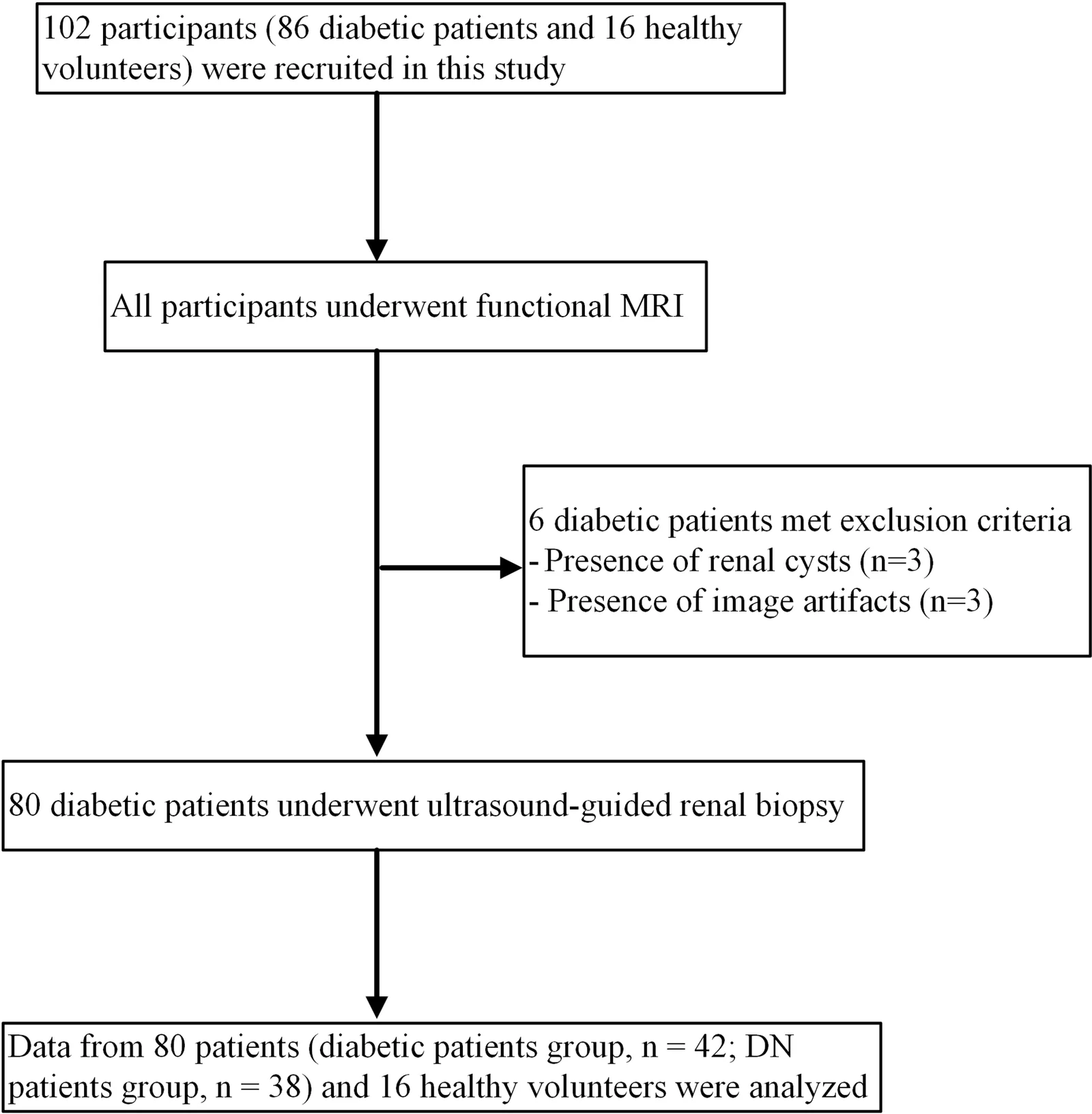
Study flowchart

**Fig. 2:**
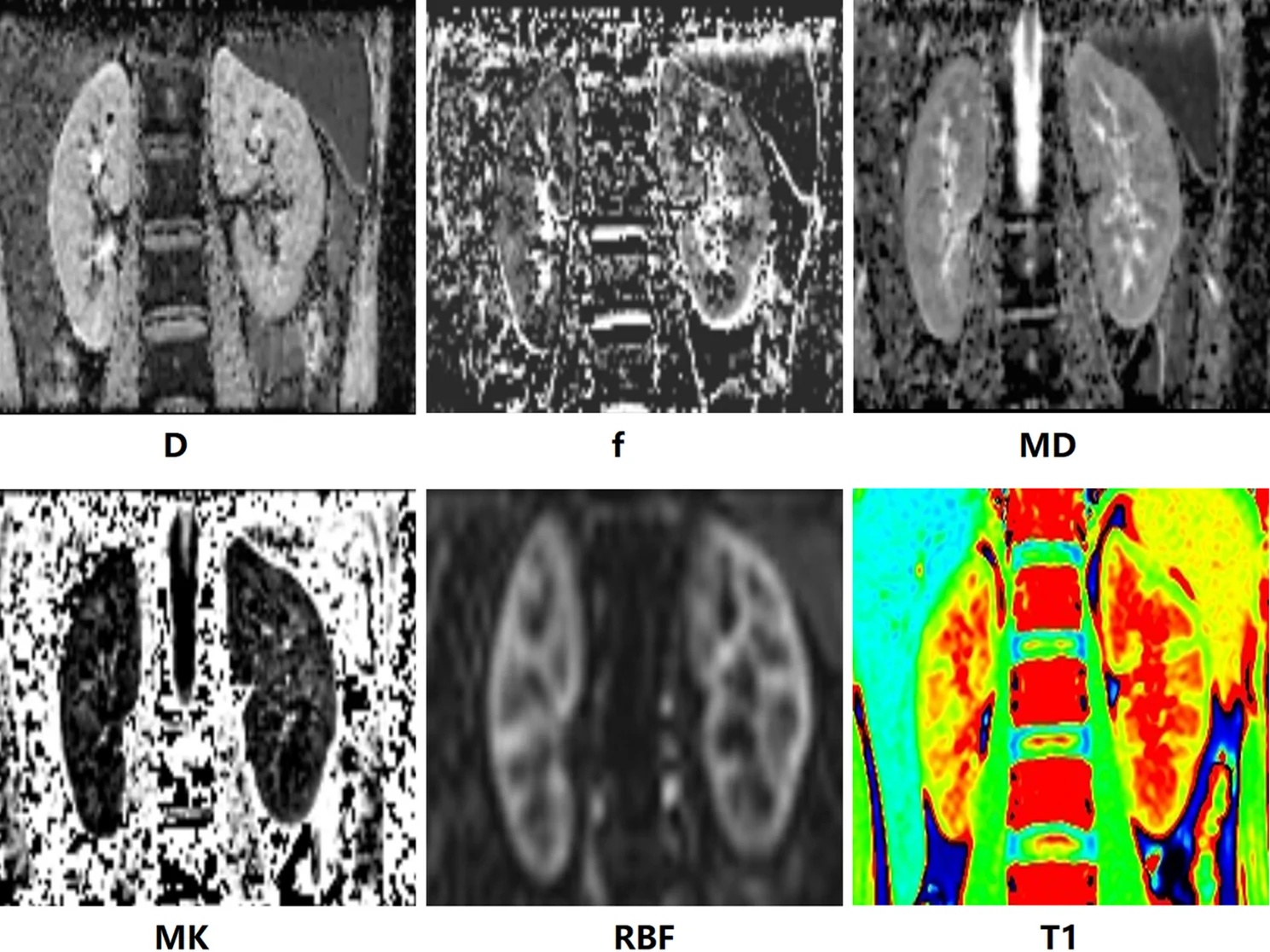
Typical examples of IVIM-DWI, DKI, ASL and T1 mapping parameters maps (healthy volunteer, 39-year-old female, eGFR: 112 mL/min/1.73 m^2^, Serum creatinine: 50µmol/L, 24 h-UA: 14.6 mg/24 h, FBG: 5.2 mmol/L, renal cortical D value: 1.68 × 10^− 3^mm^2^/s, renal cortical f value: 27.8%, renal cortical MD value: 2.73 × 10^− 3^mm^2^/s, renal cortical MK value: 0.52, renal cortical RBF value: 241.7mL/100 g/ min, renal cortical T1 value: 1320.6ms

Of the 102 participants recruited, three DP with renal cysts and three DP with image artifacts were excluded from study. Therefore, 80 patients (DP group, *n* = 42, 53.4 ± 10.0 years old; DN patients group, *n* = 38, 47.9 ± 10.9 years old) and 16 healthy volunteers (control group, 49.5 ± 9.5 years old) were ultimately included (Fig. 1). fMRI was successfully performed in all participants, and example images are depicted in Fig. 2. No significant differences in age, sex, eGFR, SCr, 24 h-UA were noted among the three groups (*P* > 0.050). As expected, both DP and DN patients had significantly higher FBG than control group (*P* < 0. 001). However, no significant differences in FBG were observed between DP and DN patients groups (*P* > 0.050).

### Comparison of MRI parameters between healthy volunteers and patients

**Fig. 3:**
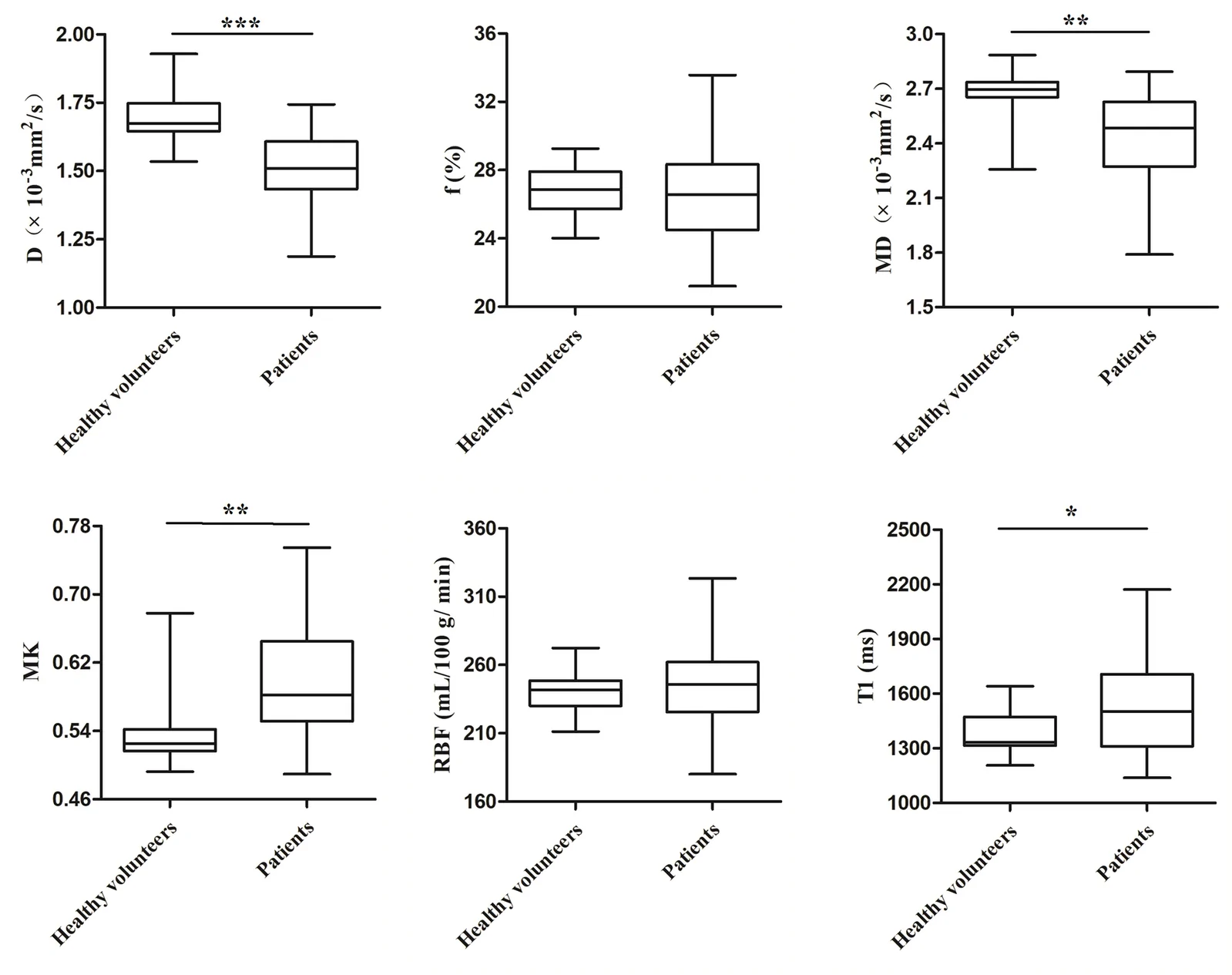
Comparison of renal cortical D, f, MD, MK, RBF and T1 values between healthy volunteers and patients (* = *p* < 0.05, **=*p* < 0.01, ***=*p* < 0.001)

The ICC of the cortical D, f, MD, MK, RBF and T1 values were 0.954 (95% confidence interval [CI], 0.905–0.978), 0.965 (0.929–0.983), 0.972 (0.943–0.987), 0.966 (0.910–0.985), 0. 969 (0.937–0.985), and 0.942 (0.883–0.972), respectively. Compared with patients, the control group had significantly higher RC D and MD values, and significantly lower MK and T1 values (*P* < 0. 001, *P* = 0.001, *P* = 0.001, *P* = 0. 020 respectively). However, RC f and RBF values didn’t significantly differ between control group and patients (*P* > 0.050) (Fig. 3).

### Comparison of MRI parameters among the three groups

**Fig. 4:**
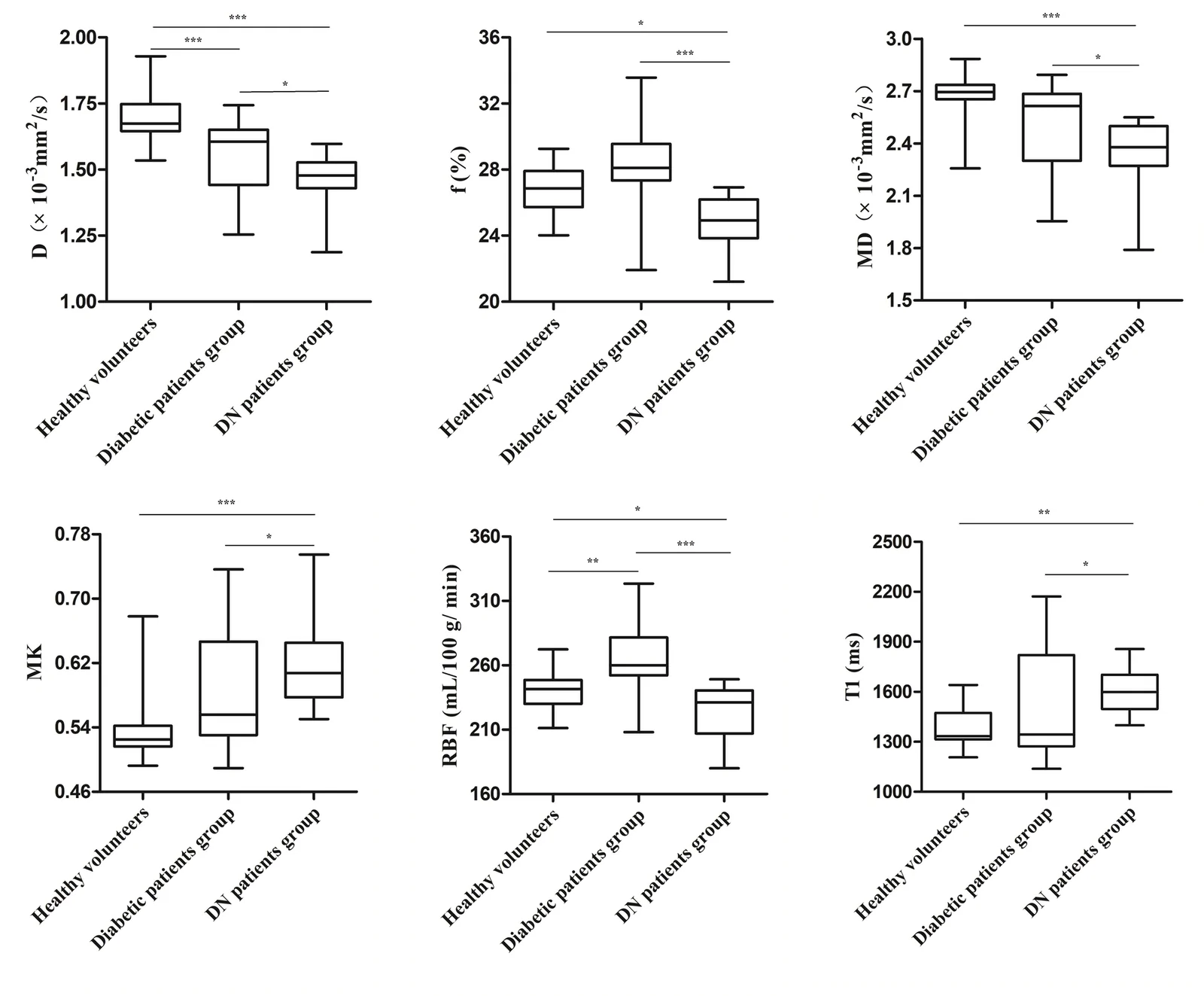
Distributions of renal cortical D, f, MD, MK, RBF and T1 values of healthy volunteers, diabetic patients group, and diabetic nephropathy (DN) patients group (* = *p* < 0.05, **=*p* < 0.01, ***=*p* < 0.001)

RC MRI parameters showed no significant difference when comparing the right kidney with the left kidney (*P* > 0.050). The RC MRI parameters exhibited statistically significant differences among three groups (*P* < 0.050). As shown in Fig. 4; Table 3, the values of RC D, f, MD, and RBF in the DN patient group were notably lower, while the values of MK and T1 were significantly higher, compared with those in the DP group and the control group (*P* < 0.050). Furthermore, the control group had a significantly higher RC D value and a significantly lower RBF value than the DP group (*P* < 0. 001, *P* = 0.002 respectively). However, no significant difference was observed in RC f, MD, MK, and T1 values between the control group and the DP group (*P* > 0.050).

**Table 3:** The renal cortical MRI parameters of the participants

	Control group	Diabetic patients group	DN patients group	*P* value	*P* ^1^ value	*P* ^2^ value	*P* ^3^ value
D (×10^− 3^mm^2^/s)	1.69 ± 0.10	1.55 ± 0.13	1.48 ± 0.08	< 0.001	< 0.001	< 0.001	0.011
f (%)	26.8 ± 1.5	28.2 ± 2.8	24.9 ± 1.5	< 0.001	0.085	0.010	< 0.001
MD (×10^− 3^mm^2^/s)	2.65 ± 0.17	2.50 ± 0.27	2.37 ± 0.15	< 0.001	0.057	< 0.001	0.017
MK	0.54 ± 0.05	0.58 ± 0.06	0.61 ± 0.04	< 0.001	0.073	< 0.001	0.011
RBF (mL/100 g/ min)	240.48 ± 16.07	262.61 ± 25.07	224.87 ± 19.48	< 0.001	0.002	0.046	< 0.001
T1 (ms)	1386.93 ± 126.43	1479.64 ± 310.39	1601.83 ± 128.26	0.004	0.351	0.006	0.048

DN: diabetic nephropathy; D: true diffusion coefficient; f: perfusion fraction; MD: mean diffusivity; MK: mean kurtosis; RBF: renal blood flow; ^1^ Post hoc paired comparisons between the control group and diabetic patients group, ^2^ Post hoc paired comparisons between the control group and DN patients group, ^3^ Post hoc paired comparisons between the diabetic patients group and DN patients group

### Correlations between MRI parameters and biochemical indexes

A negative correlation of 24 h-UA with RC D (*r* = − 0.541), f (*r* = − 0.570), MD (*r* = − 0.630), RBF (*r* = − 0.509) values, as well as of eGFR with MK (*r* = − 0.553), T1 (*r* = − 0.612) values was observed. Moreover, a positive correlation of eGFR with RC D (*r* = 0.535), f (*r* = 0.632), MD (*r* = 0.615), RBF (*r* = 0.652) values, as well as of 24 h-UA with MK (*r* = 0.550), T1 (*r* = 0.609) values was observed. It is worth noting that positive correlations were found: one between RC D and MD values (*r* = 0.722), another between f and RBF values (*r* = 0.791), and a third between MK and T1 values (*r* = 0.759) (all *P* < 0. 001).

### Diagnostic performance of MRI parameters in DN compared with biochemical indexes

**Fig. 5:**
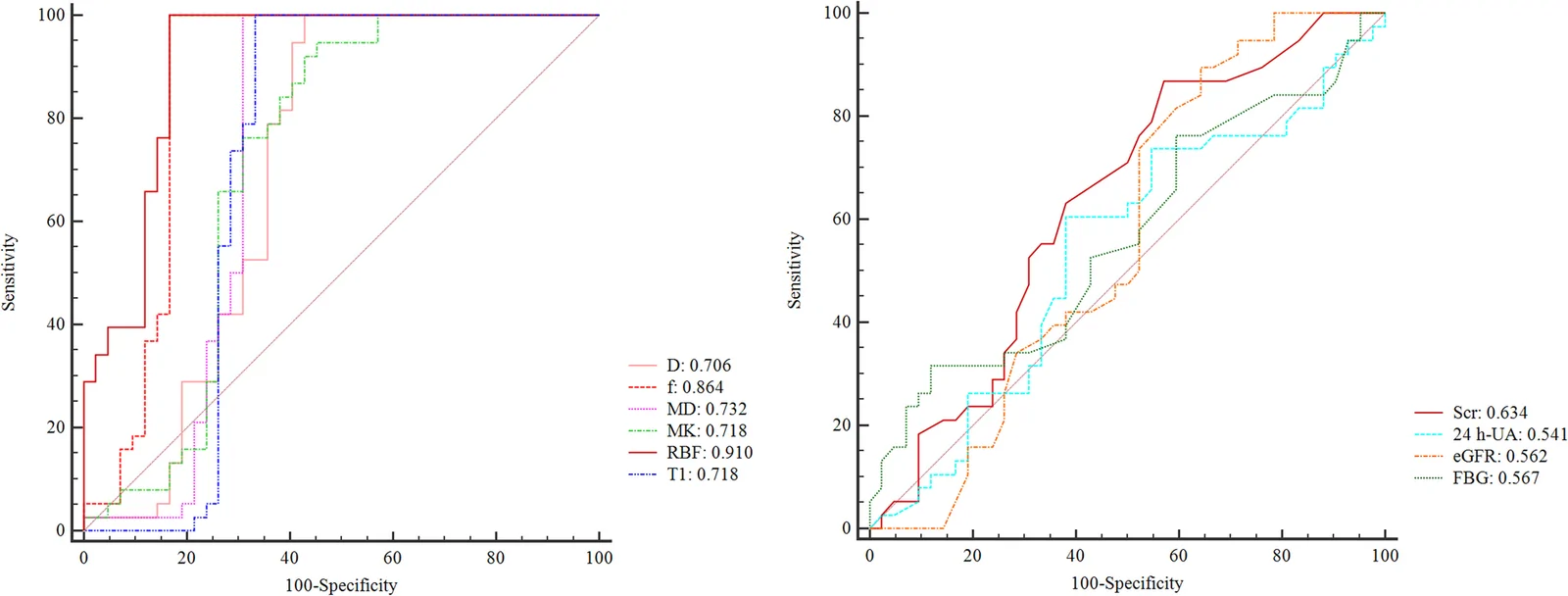
Receiver operating characteristic curves for the discrimination of diabetic nephropathy patients from the diabetic patients

For discriminating DP from DN patients groups, the AUC of eGFR, SCr, 24 h-UA and FBG were 0.562 (95% CI, 0.447–0.673), 0.634 (0.519–0.739), 0.541 (0.426–0.653), 0.567 (0.451–0.677), respectively. Additionally, RC MRI parameters could distinguish DP from DN patients groups with an AUC that ranged between 0.706 and 0.910. By contrast, the optimal parameter was identified as the RC RBF value, which gave an AUC of 0.910 (0.825–0.963), specificity of 83.3% (68.6–93.0%), and sensitivity of 100.0% (90.7–100.0%) under an optimal cut-off value of 249.1 mL/100 g/min. Subsequent analysis revealed that AUCs of RC f, RBF values were significantly larger than those of eGFR, SCr, 24 h-UA, FBG and RC D, MD, MK, T1 values (*P* < 0.050). However, there was no statistically significant difference between the AUC of the RC RBF value and that of the f value (*P* > 0.050). Detailed diagnostic test metrics for detecting DN are provided in Table 4; Fig. 5.

**Table 4:** Diagnostic performance of MRI parameters in diabetic nephropathy compared with biochemical indexes

Comparison	Cut-off value	AUC (95%CI)	Sensitivity (%) (95%CI)	Specificity (%) (95%CI)	P
Diabetic patients group (n=42) vs DN patients group (n=38)					
D (×10^-3^mm^2^/s)	≤1.60	0.706(0.594-0.803)	100.0(90.7-100.0)	57.1(41.0-72.3)	<0.001^a^
f (%)	≤26.9	0.864(0.769-0.930)	100.0(90.7-100.0)	83.3(68.6-93.0)	<0.001^b^
MD (×10^-3^mm^2^/s)	≤2.55	0.732(0.622-0.825)	100.0(90.7-100.0)	69.1(52.9-82.4)	<0.001^c^
MK	>0.56	0.718(0.606-0.813)	94.7(82.3-99.4)	54.8(38.7-70.2)	<0.001^d^
RBF (mL/100 g/ min)	≤249.10	0.910(0.825-0.963)	100.0(90.7-100.0)	83.3(68.6-93.0)	<0.001^e^
T1 (ms)	>1390.70	0.718(0.606-0.813)	100.0(90.7-100.0)	66.7(50.5-80.4)	<0.001^f^
SCr (μmol/L)	>41.00	0.634(0.519-0.739)	86.8(71.9-95.6)	42.9(27.7-59.0)	<0.001^g^
24 h-UA (mg/24 h)	>19.90	0.541(0.426-0.653)	60.5(43.4-76.0)	61.9(45.6-76.4)	<0.001^h^
eGFR (mL/min/1.73 m^2^)	≤118.00	0.562(0.447-0.673)	89.5(75.2-97.1)	35.7(21.6-52.0)	0.262^i^
FBG (mmol/L)	>7.5	0.567(0.451-0.677)	31.6(17.5-48.7)	88.1(74.4-96.0)	

95%CI: 95% confidence intervals; AUC: area under curve; DN: diabetic nephropathy; D: true diffusion coefficient; f: perfusionfraction; MD: mean diffusivity; MK: mean kurtosis; RBF: renal blood flow; eGFR: estimated glomerular filtration rate;24 h-UA: 24-hour urine albumin; FBG: fasting blood glucose; SCr: serum creatinine

^a^f vs SCr;^b^f vs 24 h-UA; ^c^f vs eGFR; ^d^f vs FBG; ^e^RBF vs SCr; ^f^RBF vs 24 h-UA; ^g^RBF vs eGFR; ^h^RBF vs FBG; ^i^RBF vs f

## Discussion

Previous study has confirmed that renal injury is reversible in early stage of DN [23], early diagnosis is essential in formulating appropriate treatment plans and improving patient prognosis. Currently, although detection of urinary microalbumin is widely used for DN diagnosis, its sensitivity in detecting early DN is low [24]. Additionally, although renal biopsy is gold standard for DN diagnosis, its widespread application is limited by its invasiveness. Previous studies have demonstrated the feasibility, scientific value and application prospects of MRI in the evaluation of diabetic kidney disease [25, 26]. In this study, we evaluated the value of multiparametric fMRI in detecting early DN in patients with type 2 diabetes compared with healthy controls. The results of this study demonstrated that IVIM-DWI, DKI, ASL, and T1 mapping allowed noninvasive detection of early DN in type 2 DP. Moreover, RC f, RBF values were more effective than biochemical indexes (eGFR, SCr, 24 h-UA, FBG) in distinguishing DP from DN patients groups. This result indicated that IVIM-DWI and ASL had the potential to serve as useful imaging tools for detecting early DN.

The detection of early DN is beneficial for slowing the progression of DN and improving treatment or patient outcomes. Our study results showed that 47.5% of DP had DN confirmed via renal biopsy. Additionally, between the DP and DN patients groups, no significant difference in eGFR, SCr, 24 h-UA and FBG was found. From this result, it can be inferred that routine clinical biochemical markers have limited value in the identification of early DN. In contrast, when compared with DP, DN patients showed a significant decrease in RC D, f, MD, and RBF values—along with a significant increase in RC MK and T1 values. This indicates that multiparametric fMRI could act as supplementary tools for early DN screening in type 2 DP.

In a previous study that assessed the utility of IVIM-DWI for identifying early renal function changes in diabetic, the DP were categorized into two groups: NAU (normal to mildly increased albuminuria) and MAU (moderately increased albuminuria) groups [27]. By contrast, we believe that the group criteria of our study more accurately represented early DN. In our study, we observed that RC D and f values in the DN patients group were significantly lower than those in DP and control groups, the DP group had a significantly lower RC D value than the control group. From the control group to the DP group and DN patients group, the RC D value shows a progressive decline, this decline signifies that water molecule diffusion in the renal cortex is constrained. Some early histologic changes may be the reason for this phenomenon, including tubular epithelial cell swelling, glomerular basement membrane thickening, mesangial expansion [28–31]. These histologic changes may lead to a reduction in extracellular space and water content, which in turn restricts the diffusion of water molecules [20]. Comparing with control group, the RC f value was slightly increased in DP and significantly decreased in DN patients groups. The increased RC f value well reflects renal hyperfiltration and hyperperfusion in DP, while the renal perfusion would decrease in DN patients.

Previous studies on animals have demonstrated the potential of DKI to assess early changes in renal function impairment in diabetes and to detect early DN [32, 33]. In our study, we found that RC MD value decreased significantly (RC MK value increased significantly) in DN patients group compared with DP and control groups. The histologic mechanism of decrease of RC MD value in DN patients was similar to that of RC D value. We found that there is a positive correlation between RC D and MD values (*r* = 0.722), which is a indication of the favorable consistency of them. The MK reflects deviation degree of water molecule diffusion from Gaussian distribution and could be used to evaluate complexity of microstructural environment of tissue [6, 34]. We speculated that the increase in RC MK value was attributed to histologic changes, including tubular epithelial cell swelling, glomerular basement membrane thickening, mesangial expansion [28–31], which would aggravate the complexity of microstructural environment of renal tissue.

A previous study demonstrated the feasibility of ASL in evaluating renal perfusion in DN [35]. In our study, comparing with control group, the RC RBF value significantly increased in DP and significantly decreased in DN patients groups. From control to DP and DN patients groups, the variation trend of RC RBF value was similar to that of f value, which may be explained by the positive correlation between RC RBF and f values (*r* = 0.791) in our study. We speculated that the increase in RC RBF value was attributed to hyperfiltration and hyperperfusion, which were caused by high blood sugar, high-protein diet and glomerular hypertrophy in DP [36, 37]. In DN patients, on the one hand, tubular epithelial cell swelling would lead to decreased tubular reabsorption, which results in decreased blood volume, and subsequently leads to decreased glomerular filtration rate and renal perfusion; on the other hand, glomerular basement membrane thickening would affect filtration function of glomerulus, resulting in reduced renal perfusion. The 2 aforementioned factors were responsible for the decrease in RC f and RBF values in DN patients. It is worth noting that the variation trend of eGFR was analogous to that of RC f and RBF values in our study. However, no significant difference in eGFR was observed among three groups. Therefore, we speculated that RC f and RBF values could reflect changes in renal blood perfusion more sensitively than eGFR.

Furthermore, in our study, we observed that RC T1 value significantly increased in DN patients group compared with DP and control groups. The native T1 value was primarily associated with the microstructural environment of tissues [38]. The histologic changes of early DN, including tubular epithelial cell swelling, glomerular basement membrane thickening, mesangial expansion, would aggravate the complexity of microstructural environment of renal tissue, and subsequently leads to increased RC T1 value.

A prior study that assesses the utility of IVIM-DWI in detecting early renal function changes in diabetics observed that RC D value was correlated with urinary albumin-creatinine ratio (ACR) and eGFR [27]. Furthermore, a previous study investigating value of ASL to detect early hemodynamic changes in DN observed a correlation between RC RBF value and eGFR [35]. In our study, RC MRI parameters significantly correlated with eGFR, 24 h-UA. The findings of our study indicated that IVIM-DWI, DKI, ASL, and T1 mapping can serve as an effective tool for assessing renal injury in DN.

ROC curve analysis provided support for the use of IVIM-DWI, DKI, ASL, and T1 mapping in distinguishing DN patients from DP, with high sensitivity and a large AUC serving as certification for this application. Of greater importance, our study showed that AUCs of RC f, RBF values were statistically greater than those of eGFR, SCr, 24 h-UA, FBG for discrimination between DN patients and DP groups, in contrast, the diagnostic efficacy of eGFR, SCr, 24 h-UA, FBG was low. As a result, we hold the view that IVIM-DWI and ASL may have greater effectiveness in identifying DN in DP with normal eGFR and 24 h-UA.

We applied a free-breathing acquisition combined with motion correction strategies to IVIM-DWI, DKI, and ASL in our study, and we believed this method was more appropriate than respiratory triggering and breath-holding technology. This could be attributed to the advantages of free-breathing acquisition: it outperformed the respiratory triggering scheme in terms of time efficiency, and was more comfortable for patients than the breath-holding sequence—an advantage that was especially notable for the elderly, and the free-breathing acquisition can be useful for children in other applications/studies. Additionally, our study found that various fMRI parameters had high test-retest reliability, which demonstrated that data and results of our study were reliable.

Our study was subject to limitations that were inherent in clinical research involving human participants. Firstly, since this study’s sample size was comparatively small and it was limited to a single center, the findings of our study were preliminary, multicenter studies that have a larger sample size should be implemented in the future. Secondly, the ROI was manually drawn to measure MRI parameters, which might bring subjectivity errors or bias. However, we believed these could be reduced to a minimum, seeing as diabetes affects the entire kidney. Thirdly, a fixed PLD was used to maintain scanning consistency. Nevertheless, patients with altered renal blood flow usually exhibit changed arterial transit time, and an optimized or patient-specific PLD would be more appropriate to minimize perfusion quantification bias. Further protocol optimization with multiple PLD measurements will be conducted in subsequent research to achieve more reliable renal perfusion evaluation. Finally, our study only assessed renal cortical MRI parameters, which may compromise the comprehensiveness of our conclusions. Further research will be conducted in the future to explore the value of renal medullary MRI parameters in evaluating diabetic nephropathy.

## Conclusions

Ultimately, our study showed that multiparametric fMRI could function as a potentially valuable tool for assessing renal function in DP. Additionally, IVIM-DWI and ASL displayed substantial promise as noninvasive tools to detect DN in high-risk patients with type 2 diabetes.

## Data Availability

The datasets generated and/or analysed during the current study are not publicly available due to patient privacy regulations but are available from the corresponding author upon reasonable request.

## Abbreviations

ACR: Albumin-creatinine ratio
ASL: Arterial spin labeling
AUC: Area under the receiver operating characteristic curve
CI: Confidence interval
D: True diffusion coefficient
DKI: Diffusion kurtosis imaging
DN: Diabetic nephropathy
DP: Diabetic patients
eGFR: estimated glomerular filtration rate
f: Perfusion fraction
FBG: Fasting blood glucose
fMRI: functional MRI
24 h-UA: 24-hour urine albumin
ICC: Intraclass correlation coefficients
IVIM-DWI: Intravoxel incoherent motion diffusion-weighted imaging
MD: Mean diffusivity
MK: Mean kurtosis
pCASL: pseudo-continuous arterial spin labeling
PLD: Post-labeling delay
RBF: Renal blood flow
RC: Renal cortical
ROC: Receiver operating characteristic
ROIs: Regions of interest
SCr: Serum creatinine

## References

[1] Jha V, Garcia-Garcia G, Iseki K, Li Z, Naicker S, Plattner B, Saran R, Wang AY, Yang CW. Chronic kidney disease: global dimension and perspectives. Lancet. 2013;382(9888):260–72. 10.1016/S0140-6736(13)60687-X 23727169

[2] Macisaac RJ, Ekinci EI, Jerums G. Markers of and risk factors for the development and progression of diabetic kidney disease. AM J KIDNEY DIS. 2014;63(2 Suppl 2):S39–62. 10.1053/j.ajkd.2013.10.048 24461729

[3] Mogensen CE. Early glomerular hyperfiltration in insulin-dependent diabetics and late nephropathy. SCAND J CLIN LAB INV. 1986;46(3):201–6. 10.3109/00365518609083660 3715374

[4] Herman-Edelstein M, Scherzer P, Tobar A, Levi M, Gafter U. Altered renal lipid metabolism and renal lipid accumulation in human diabetic nephropathy. J LIPID RES. 2014;55(3):561–72. 10.1194/jlr.P040501 PMC3934740 24371263

[5] Le Bihan D, Breton E, Lallemand D, Grenier P, Cabanis E, Laval-Jeantet M. MR imaging of intravoxel incoherent motions: application to diffusion and perfusion in neurologic disorders. Radiology. 1986;161(2):401–7. 10.1148/radiology.161.2.3763909 3763909

[6] Jensen JH, Helpern JA. MRI quantification of non-Gaussian water diffusion by kurtosis analysis. NMR BIOMED. 2010;23(7):698–710. 10.1002/nbm.1518 PMC2997680 20632416

[7] Cutajar M, Thomas DL, Hales PW, Banks T, Clark CA, Gordon I. Comparison of ASL and DCE MRI for the non-invasive measurement of renal blood flow: quantification and reproducibility. EUR RADIOL. 2014;24(6):1300–8. 10.1007/s00330-014-3130-0 24599625

[8] Cox EF, Buchanan CE, Bradley CR, Prestwich B, Mahmoud H, Taal M, Selby NM, Francis ST. Multiparametric Renal Magnetic Resonance Imaging: Validation, Interventions, and Alterations in Chronic Kidney Disease. FRONT PHYSIOL. 2017;8:696. 10.3389/fphys.2017.00696 PMC5603702 28959212

[9] Lu F, Yang J, Yang S, Bernd K, Fu C, Yang C, Xu H, Liu M, Zhan S, Wang C, et al. Use of Three-Dimensional Arterial Spin Labeling to Evaluate Renal Perfusion in Patients With Chronic Kidney Disease. J MAGN RESON IMAGING. 2021;54(4):1152–63. 10.1002/jmri.27609 33769645

[10] Pi S, Li Y, Lin C, Li G, Wen H, Peng H, Wang J. Arterial spin labeling and diffusion-weighted MR imaging: quantitative assessment of renal pathological injury in chronic kidney disease. ABDOM RADIOL. 2023;48(3):999–1010. 10.1007/s00261-022-03770-4 36598569

[11] Hua C, Qiu L, Zhou L, Zhuang Y, Cai T, Xu B, Hao S, Fang X, Wang L, Jiang H. Value of multiparametric magnetic resonance imaging for evaluating chronic kidney disease and renal fibrosis. EUR RADIOL. 2023;33(8):5211–21. 10.1007/s00330-023-09674-1 37148348

[12] Lin J, Zhu C, Cui F, Qu H, Zhang Y, Le X, Yin J, Cao Y. Based on functional and histopathological correlations: is diffusion kurtosis imaging valuable for noninvasive assessment of renal damage in early-stage of chronic kidney disease? INT UROL NEPHROL. 2024;56(1):263–73. 10.1007/s11255-023-03632-y 37326823

[13] Levey AS, Stevens LA, Schmid CH, Zhang YL, Castro AR, Feldman HI, Kusek JW, Eggers P, Van Lente F, Greene T, et al. A new equation to estimate glomerular filtration rate. ANN INTERN MED. 2009;150(9):604–12. 10.7326/0003-4819-150-9-200905050-00006 PMC2763564 19414839

[14] Kordbacheh H, Seethamraju RT, Weiland E, Kiefer B, Nickel MD, Chulroek T, Cecconi M, Baliyan V, Harisinghani MG. Image quality and diagnostic accuracy of complex-averaged high b value images in diffusion-weighted MRI of prostate cancer. Abdom Radiol (New York). 2019;44(6):2244–53. 10.1007/s00261-019-01961-0 30838425

[15] Robson PM, Madhuranthakam AJ, Dai W, Pedrosa I, Rofsky NM, Alsop DC. Strategies for reducing respiratory motion artifacts in renal perfusion imaging with arterial spin labeling. MAGN RESON MED. 2009;61(6):1374–87. 10.1002/mrm.21960 PMC2946256 19319891

[16] Zhang Y, Kapur P, Yuan Q, Xi Y, Carvo I, Signoretti S, Dimitrov I, Cadeddu JA, Margulis V, Muradyan N, et al. Tumor Vascularity in Renal Masses: Correlation of Arterial Spin-Labeled and Dynamic Contrast-Enhanced Magnetic Resonance Imaging Assessments. CLIN GENITOURIN CANC. 2016;14(1):e25–36. 10.1016/j.clgc.2015.08.007 PMC4698181 26422014

[17] Bones IK, Harteveld AA, Franklin SL, van Osch MJP, Hendrikse J, Moonen CTW, Bos C, van Stralen M. Enabling free-breathing background suppressed renal pCASL using fat imaging and retrospective motion correction. MAGN RESON MED. 2019;82(1):276–88. 10.1002/mrm.27723 PMC6593735 30883873

[18] Le Bihan D, Breton E, Lallemand D, Aubin ML, Vignaud J, Laval-Jeantet M. Separation of diffusion and perfusion in intravoxel incoherent motion MR imaging. Radiology. 1988;168(2):497–505. 10.1148/radiology.168.2.3393671 3393671

[19] Jensen JH, Helpern JA, Ramani A, Lu H, Kaczynski K. Diffusional kurtosis imaging: the quantification of non-gaussian water diffusion by means of magnetic resonance imaging. MAGN RESON MED. 2005;53(6):1432–40. 10.1002/mrm.20508 15906300

[20] Li Q, Li J, Zhang L, Chen Y, Zhang M, Yan F. Diffusion-weighted imaging in assessing renal pathology of chronic kidney disease: A preliminary clinical study. EUR J RADIOL. 2014;83(5):756–62. 10.1016/j.ejrad.2014.01.024 24581595

[21] Tervaert TW, Mooyaart AL, Amann K, Cohen AH, Cook HT, Drachenberg CB, Ferrario F, Fogo AB, Haas M, de Heer E, et al. Pathologic classification of diabetic nephropathy. J AM SOC NEPHROL. 2010;21(4):556–63. 10.1681/ASN.2010010010 20167701

[22] DeLong ER, DeLong DM, Clarke-Pearson DL. Comparing the areas under two or more correlated receiver operating characteristic curves: a nonparametric approach. Biometrics. 1988;44(3):837–45. 3203132

[23] Atkins RC, Polkinghorne KR, Briganti EM, Shaw JE, Zimmet PZ, Chadban SJ. Prevalence of albuminuria in Australia: the AusDiab Kidney Study. KIDNEY INT SUPPL. 2004;92:S22–4. 10.1111/j.1523-1755.2004.09206.x 15485411

[24] Karimi F, Moazamfard M, Taghvaeefar R, Sohrabipour S, Dehghani A, Azizi R, Dinarvand N. Early Detection of Diabetic Nephropathy Based on Urinary and Serum Biomarkers: An Updated Systematic Review. ADV BIOMED RES-INDIA. 2024;13:104. 10.4103/abr.abr_461_23 PMC11665175 39717256

[25] Makvandi K, Hockings PD, Jensen G, Unnerstall T, Leonhardt H, Jarl LV, Englund C, Francis S, Sundgren AK, Hulthe J, et al. Multiparametric magnetic resonance imaging allows non-invasive functional and structural evaluation of diabetic kidney disease. CLIN KIDNEY J. 2022;15(7):1387–402. 10.1093/ckj/sfac054 PMC9217657 35756740

[26] Friedli I, Baid-Agrawal S, Unwin R, Morell A, Johansson L, Hockings PD. Magnetic Resonance Imaging in Clinical Trials of Diabetic Kidney Disease. J CLIN MED. 2023;12(14):4625. 10.3390/jcm12144625 PMC10380287 37510740

[27] Feng YZ, Chen XQ, Yu J, Liu XL, Cheng ZY, Ren WW, Feng L, Cai XR. Intravoxel incoherent motion (IVIM) at 3.0 T: evaluation of early renal function changes in type 2 diabetic patients. ABDOM RADIOL. 2018;43(10):2764–73. 10.1007/s00261-018-1555-7 29525883

[28] Luo B, Wen S, Chen YC, Cui Y, Gao FB, Yao YY, Ju SH, Teng GJ. LOX-1-Targeted Iron Oxide Nanoparticles Detect Early Diabetic Nephropathy in db/db Mice. MOL IMAGING BIOL. 2015;17(5):652–60. 10.1007/s11307-015-0829-5 25666291

[29] Xiao L, Zhu X, Yang S, Liu F, Zhou Z, Zhan M, Xie P, Zhang D, Li J, Song P, et al. Rap1 ameliorates renal tubular injury in diabetic nephropathy. Diabetes. 2014;63(4):1366–80. 10.2337/db13-1412 PMC3964498 24353183

[30] Peng XG, Bai YY, Fang F, Wang XY, Mao H, Teng GJ, Ju S. Renal lipids and oxygenation in diabetic mice: noninvasive quantification with MR imaging. Radiology. 2013;269(3):748–57. 10.1148/radiol.13122860 23901127

[31] Bakris GL. Microalbuminuria: prognostic implications. CURR OPIN NEPHROL HY. 1996;5(3):219–23. 10.1097/00041552-199605000-00006 8737856

[32] Zhou H, Zhang J, Zhang XM, Chen T, Hu J, Jing Z, Jian S. Noninvasive evaluation of early diabetic nephropathy using diffusion kurtosis imaging: an experimental study. EUR RADIOL. 2021;31(4):2281–8. 10.1007/s00330-020-07322-6 32997177

[33] Cheng ZY, Chen PK, Feng YZ, Chen XQ, Qian L, Cai XR. Preliminary Feasibility Study on Diffusion Kurtosis Imaging to Monitor the Early Functional Alterations of Kidneys in Streptozocin-Induced Diabetic Rats. ACAD RADIOL. 2023;30(8):1544–51. 10.1016/j.acra.2022.09.016 36244869

[34] Raab P, Hattingen E, Franz K, Zanella FE, Lanfermann H. Cerebral gliomas: diffusional kurtosis imaging analysis of microstructural differences. Radiology. 2010;254(3):876–81. 10.1148/radiol.09090819 20089718

[35] Mora-Gutierrez JM, Garcia-Fernandez N, Slon RM, Paramo JA, Escalada FJ, Wang DJ, Benito A, Fernandez-Seara MA. Arterial spin labeling MRI is able to detect early hemodynamic changes in diabetic nephropathy. J MAGN RESON IMAGING. 2017;46(6):1810–7. 10.1002/jmri.25717 28383796

[36] Caramori ML, Fioretto P, Mauer M. Low glomerular filtration rate in normoalbuminuric type 1 diabetic patients: an indicator of more advanced glomerular lesions. Diabetes. 2003;52(4):1036–40. 10.2337/diabetes.52.4.1036 12663477

[37] Su J, Li SJ, Chen ZH, Zeng CH, Zhou H, Li LS, Liu ZH. Evaluation of podocyte lesion in patients with diabetic nephropathy: Wilms’ tumor-1 protein used as a podocyte marker. DIABETES RES CLIN PR. 2010;87(2):167–75. 10.1016/j.diabres.2009.10.022 19969384

[38] Graham-Brown MP, Singh A, Wormleighton J, Brunskill NJ, McCann GP, Barratt J, Burton JO, Xu G. Association between native T1 mapping of the kidney and renal fibrosis in patients with IgA nephropathy. BMC NEPHROL. 2019;20(1):256. 10.1186/s12882-019-1447-2 PMC6621982 31296183

